# Transcatheter closure of patent ductus arteriosus in preterm ventilation-dependent neonates: A case series report

**DOI:** 10.1097/MD.0000000000022528

**Published:** 2020-11-20

**Authors:** Xiaoqing Shi, Yimin Hua, Yifei Li

**Affiliations:** Key Laboratory of Birth Defects and Related Diseases of Women and Children of MOE, Department of Pediatrics, West China Second University Hospital, Sichuan University, Chengdu, China.

**Keywords:** hsPDA, premature neonate, transcatheter closure, ventilation-dependent

## Abstract

**Objective:**

Surgical closure of patent ductus arteriosus (PDA) has been considered the only way to rescue preterm neonates following nonsteroidal anti-inflammatory drugs closure failure. However, PDA closure by catheter-based interventions has become another therapeutic choice. The aim of this report was to investigate the timing and treatment methods for hemodynamically significant PDA (hsPDA) in preterm neonates.

**Methods:**

We retrospectively studied 4 ventilator-dependent preterm neonate cases with hsPDA who had an urgent need of PDA closure and who attended our hospital between October 2016 and March 2018. We assessed the efficacy and safety of transcatheter closure of the hsPDA, and evaluated the dependence of the infants on mechanical ventilation.

**Results:**

The 4 infants with hsPDA underwent successful transcatheter closures. Two infants were weaned from the ventilatory support within 24 hours after the closure. Those 2 preterm neonates demonstrated normal growth and development during the postoperative follow-up. However, the other 2 infants still needed ventilatory support beyond 48  hours post procedure. One of them presented a bronchial stenosis, underwent a bronchial stent placement by bronchoscopy 10 days after the PDA closure, and was only then finally withdrawn from the ventilatory support. The other infant had a severe bronchomalacia and was only weaned from the ventilator 36 days post PDA closure.

**Conclusion:**

Transcatheter closure could be an acceptable alternative to surgical ligation when medication treatment fails to close hsPDAs in ventilator-dependent preterm neonates. This intervention should be considered to minimize mechanical ventilation duration, reduce the incidence of bronchopulmonary dysplasia, and improve the prognoses of these infants.

## 1 Introduction

Persistent opening of the ductus arteriosus is common in preterm neonates resulting in patent ductus arteriosus (PDA). Hemodynamically significant PDA (hsPDA) induces cardiac dysfunction and prolongs mechanical ventilation in premature neonates, leading to bronchopulmonary dysplasia (BPD), neurological development delays, retinopathy of prematurity, necrotizing enterocolitis, other complications, and even death. Interventions, including pharmacological drug administration, surgical ligation, and transcatheter closure, need to be appropriately timed to improve the prognosis and quality of life of these patients.^[1–3]^

Surgical closure of PDA used to be the only way to rescue preterm neonates following nonsteroidal anti-inflammatory drug-mediated closure failure or drug-ingestion contraindications. However, catheter-based interventions for PDA closure have become an alternative therapeutic choice.^[4]^ Few studies have focused on the application criteria and the follow-up outcomes of ventilator-dependent preterm neonates with heart dysfunction undergoing transcatheter PDA closure.^[5,6]^ Herein, we report a retrospective case analysis of 4 ventilator-dependent preterm neonates referred to our center for transcatheter PDA closure using an Amplatzer Ductus Occluder (ADO) from October 2016 to March 2018. The inform consents have been obtained from the patient's parents for describing the case details and demonstrating related radiological or echocardiographic images scientifically. This research has been approved by the ethics committee of West China Second University Hospital, Sichuan University (2014-034).

## 2 Methods

### 2.1 Patient population

This is a single-center, retrospective, consecutive, case series to analyze the efficacy and safety of transcatheter closure as an alternative to surgical ligation and medication treatment. We followed the PROCESS guidelines during this study. Four patients underwent hsPDA closure attempts using an ADO (AGA Medical, Golden Valley, Minnesota, United States of America) between October 2016 and March 2018 in the Pediatric Heart Center of the West China Second University Hospital (Sichuan University). The ethics committee of the West China Second University Hospital in Sichuan University approved this study. We obtained written consent forms from the parents of all participants. All patients were diagnosed as having PDA by transthoracic echocardiography, and the diagnoses were confirmed during the transcatheter procedure by angiography. Prior to the closure procedure, chest radiography and electrocardiography were performed to detect pulmonary and cardiovascular concerns, and angiography was used to confirm the shape and size of the PDA. We registered the study at our clinical research center, Sichuan University (number 2014-034).

### 2.2 Inclusion and exclusion criteria

We defined the following inclusion criteria:

1) presence of an ultrasound-diagnosed hsPDA (> 2.0 mm in size; pulsatile low-speed blood flow of large artery, left atrial to aortic root ratio > 1.4);
2) preterm delivery (< 36 gestational weeks);
3) mechanical ventilation applied due to cardiopulmonary dysfunction and systemic hypoperfusion-related symptoms, and 2 ventilator withdrawal failures;
4) laboratory test results showing high levels of NT-proBNP and cTnI, and inotropic drugs needed to maintain acceptable hemodynamic states;
5) failed pharmacological closure after nonsteroidal anti-inflammatory drug administration and indications for an urgent surgical ligation.

The exclusion criteria included:

1) patients presenting any other cardiac malformation; and,
2) presence of severe complications (intracerebral hemorrhage and necrotizing enterocolitis).

### 2.3 Procedure

The hsPDA transcatheter closures were performed under general anesthesia. We followed the transcatheter treatment of congenital heart disease guideline of the Chinese Pediatric Cardiology Association during the transcatheter closure procedure. Right and left cardiac catheterizations were performed via the percutaneous transfemoral route. Hemodynamics of the pulmonary vessels were monitored and pulmonary vessel resistance was assessed before the final occlusion. Echocardiography imaging was performed during the procedure to assess the profile of the PDA in the short axis section of the cardiac base oblique view. The size and the shape of the PDA were further confirmed by angiography. The size of the ADO device was selected to be 1 mm larger than the ductus measured by angiocardiography. Heparin (100 U/kg) was administered to all patients after successful femoral artery access. Aspirin was prescribed for 24 months after the procedure.

### 2.4 Outcomes and follow-up

We collected clinical baseline, closure, and postoperative follow-up data retrospectively. All patients who received an asymmetric ADO underwent follow-ups for up to 12 months. The characteristics at birth were recorded, gestational weeks, Apgar score, immediate treatment in the neonatal intensive care unit. Moreover, we obtained the information on the administration of mechanical ventilation, and that on the pharmacological therapeutic strategy. The weaning times from the ventilator were carefully stated, and the development and maintenance of tracheobronchial structures and pulmonary function were monitored during follow ups.

### 2.5 Statistical analysis

We presented the patient data according to the procedures and follow-ups because of the low number of cases in our study. Quantitative data were presented as means with ranges, while qualitative data were expressed as numbers of cases (n). We did not make comparisons due to the lack of controlled cohorts.

## 3 Results

Four preterm neonates were enrolled in this study, including 3 baby girls and 1 baby boy (Table 1). The gestational ages ranged from 29^+1^ to 32^+6^ weeks. The average age at device closure was 22 days (range, 15 to 28 days), with an average body weight of 1.76 kg (range, 1.6 to 1.9 kg). Echocardiographic images revealed a mean PDA diameter at 2.85 mm (range, 2.0 to 3.5 mm), with a mean left atrial to aortic root ratio at 1.78 (range, 1.5 to 2.0), and a pulmonary flow/systemic flow ratio at 1.98 (range, 1.6 to 2.4). The mean NT-proBNP level ranged between 341 and 970 pg/ml. All preterm neonates had PDA after 3 intravenous ibuprofen administrations (10 mg/kg for the first time, and 5 mg/kg after 24 h and 48 h). In addition, all preterm neonates received continuous infusion of dopamine (5–8 μg/kg/min) for 3 to 5 days and oral digoxin administration (6–8 μg/kg/d) to maintain stable circulatory function and systemic perfusion. The mean preoperative mechanical ventilation duration was 18.3 days (range, 12 to 24 days).

**Table 1:** The clinical characteristics of enrolled patients.

Case	Gestational weeks (Wk)	Body weight at closure (Kg)	Age at closure (d)	Heart rate (Beats per min)	Breathing (Per min)	Preoperative mechanical ventilation time (d)	BPD	PDA diameter (mm)	LA/AO	Qp/Qs	pro-BNP (pg/mL)
1	32^+1^	1.75	26	168	52	24	+	2.0	1.9	1.6	970
2	32^+2^	1.8	28	158	60	22	+	2.5	1.5	1.5	580
3	30^+4^	1.9	15	180	58	15	+	3.5	2.0	1.8	341
4	29^+1^	1.6	19	164	60	18	+	3.5	1.7	1.4	670

BPD = bronchopulmonary dysplasia, LA/AO = left Atrial to Aortic root ratio, PDA = patent ductus arteriosus, Qp/Qs = pulmonary flow/systemic flow.

According to the angiographic findings, occluders of diameter from 4/6 to 8/10 mm and transfer sheaths from 5 to 7 F were selected for transcatheter closure. The operations took 30 to 75 minutes. Post-implantation angiographic images demonstrated that occluders had been well placed at the correct position and confirmed the absence of stenoses of the descending aorta and pulmonary artery. Two preterm neonates were successfully weaned from the ventilator 24 hours after closure, and they demonstrated normal growth and development during the postoperative follow-up. One of the other 2 infants had to be withdrawn from the ventilator 22 days after the PDA closure due to the formation of bronchial stenosis; the neonate underwent a bronchial stent placement 10 days after PDA closure. The other infant presented severe bronchomalacia and was weaned from the ventilator 36 days post PDA closure (Table 2).

**Table 2:** Procedure and follow-up information.

Case	Intravenous ibuprofen	Contrast PDA diameter	Occluder size (mm)	Transfer sheath (F)	Weaning time (h)	Bronchoscopy	Follow-up
Case 1	Failed	2.0	4/6	5	24	–	Good
Case 2	Failed	3.0	6/8	6	19	–	Good
Case 3	Failed	2.4	6/8	6	Not evacuate	Bronchial stenosis	Wean ventilator after bronchial stent
Case 4	Failed	4.0	8/10	6	Not evacuate	Bronchomalacia	Bronchial asthma

PDA = patent ductus arteriosus.

## 4 Discussion

This study demonstrates that the use of ADOs for interventional therapy of ventilator-dependent preterm neonates with PDA is safe and feasible. However, 2 preterm neonates could not be weaned from the ventilator within the first 48 hours after transcatheter closure, indicating the challenge in choosing the right operative timing for these catheter-based interventions. The main reasons for the failure of those 2 ventilator-dependent cases were bronchial issues, with BPD developing after mechanical ventilation periods longer than 20 days. Studies have shown evidence of air-trapping in the expiratory high-resolution computed tomography images of BPD survivors, with a significant increase in the incidence of major airway lesions, such as tracheobronchomalacia and iatrogenic tracheobronchial stenoses.^[7–9]^ Thus, early intervention should be considered for ventilator-dependent preterm neonates with hsPDA to minimize mechanical ventilation in an effort to reduce the incidences of BPD, bronchomalacia, and bronchial stenoses and to improve their prognoses.^[10]^

PDA is an independent risk for the development of BPD in preterm neonates, whereas the hemodynamic abnormalities associated with the surgical intervention are also independent risk factors for BPD. The challenge remains in how to balance the risks between PDA-associated BPD and surgery-associated BPD. In this study, 4 ventilator-dependent premature neonates underwent transcatheter closure of hsPDA in our hospital; all interventional surgeries went well, and all air-trapping symptoms disappeared. Two infants underwent transcatheter closure at the ages of 15 and 19 days and were weaned from the ventilator within 24 hours of the procedure. However, the 2 other infants underwent closures at the ages of 26 and 28 days, but their ventilators could not be weaned after the successful procedures due to bronchomalacia in 1 and to bronchial stenosis in the other. Based on a literature review and on our preliminary experiences, the ages from 10 days to 6 weeks may be the best for performing interventions in preterm neonates with PDA. Too early an intervention (within 10 days) increases the risks of surgery and the incidence of complications, while late interventions (after 6 weeks) could lead to sustained ventilator-dependence due to BPD or tracheobronchial stenoses that may have developed before the intervention. However, more evidence is needed to confirm the most appropriate timing for transcatheter closure interventions.^[11,12]^

Meanwhile, full consideration should be given to the instrument size for interventional occlusion and to the cardiac and vascular spaces of the premature neonates with PDA for early interventions. Low body weight is still a major challenge for transcatheter occlusions in preterm neonates with PDA. In a multicenter study on the independent risks for adverse cardiac events in cardiac catheterizations and interventional therapy of infants, a multivariate analysis identified body weight < 2 kg as an independent risk for complications after transcatheter closure. Therefore, to improve the success rate of interventional closures and to reduce the incidence of postoperative complications, choosing a suitable occluder and transporter according to each circumstance is important. Currently, transcatheter PDA closures can be performed with different types of occluders, including coil, ADO, ADOII, AVPII, AVPIV, and ADOII AS devices,^[13]^ and the minimum body weights to perform closures with these occluders are 930, 2210, 2185, 870, 1860, and 1180 g, respectively. The coil is a safe and feasible choice for the closure of diameters below 1.5 to 2 mm. However, larger PDA diameters would result in residual shunts after closure, and multiple occluders would need to be placed, leading to longer operation times and higher complications risks. ADO and ADOII can be used in premature neonates with body weights of approximately 2.0 kg, but attention must be paid as severe vascular complications may occur if the PDA diameter is 3-4 mm. For these PDA diameters larger occluders and transporters need to be used (the 7-F sheathing diameter is 2.23 mm; most premature neonates with body weights around 2.0 kg have a femoral vein diameter of approximately 2.0 mm, a descending aorta diameter of 3.5 to 5.5 mm and a pulmonary artery diameter of approximately 3.0 mm-5.0 mm). In addition, the side umbrella surface of ADOs exceeds the diameter of the waist artery by approximately 4 to 6 mm, which could cause secondary descending aortic stenosis. Regarding the ADOII, although the impact on the surrounding DA structure is minimized, the size of the transporter cannot be effectively reduced due to the massive polyester fabric filler in the high density of the Ni-Ti memory alloy fabric device. In this study, 4 premature neonates with PDA had relatively heavy body weights; therefore, ADOs were chosen for transcatheter closures and the procedures were successful. AVPII and AVPIV have low fabric device densities and small amounts of polyester fabric filler, effectively minimizing the diameter of the transporter. Moreover, anastomoses with the blood vessel have been considered, particularly when designing AVPIVs. However, AVPII and AVPIV remain unfeasible for low birth-weight premature neonates with large-diameter PDAs. ADOII AS is a Ni-Ti alloy occluder that was specifically designed for PDAs with a narrow aortopulmonary artery; the umbrella of the ADOII AS is only 1 mm wider than its waist, and the length is similar to that of the AVPIV. Therefore, ADOII AS can be easily derived and recycled through a 4-F sheath. The results of both animal experiments and clinical applications have shown that ADOII AS can play an effective role, and that the transporter of ADOII AS achieves a real miniaturization. The delivery and recovery operations only require a 3- to 4-F sheath, which is feasible for transcatheter closures in low body weight and young premature neonates with PDA. According to the morphology, PDAs were divided into 6 types by Philip (conical, tubular, window, saccular, elongated, and fetal PDAs). The fetal type is the most common PDA in premature neonates causing up to 79% of them. The fetal type has a hockey-like shape, with a large, long, and tortuous diameter. The more suitable choices for closure of fetal type PDAs are the AVPIV and ADOII AS.^[14]^

## 5 Conclusion

With the elucidation of the pathogenesis of PDAs and the improvement in the diagnostic technology combined with the miniaturization of the occluder and transporter, catheter-based interventions are developing rapidly for the treatment of PDAs.^[4]^ Four premature neonates underwent transcatheter closure successfully under short operation durations. In recent years, transcatheter hsPDA closure has been considered an alternative treatment due to optimal procedure durations and delivery strategy selections. Some preliminary clinical practice cohorts and case series have been reported. However, strict indication criteria for transcatheter hsPDA closures need to be followed:

(1) the PDA should be causing abnormal hemodynamics and heart dysfunction;
(2) hyperperfusion of the cardiopulmonary system requiring ventilatory support should have been identified;
(3) the intubation-based ventilator administration should be prolonged with failed attempts to withdraw the ventilation system; and,
(4) pharmacological treatments need to have failed to close the PDA and high risk or contraindications against surgical ligation must exist.
(5) Transcatheter closure may be an alternative to surgical ligation when medication treatments have failed to close hsPDAs in ventilator-dependent preterm neonates. In addition, the procedure should be considered to minimize mechanical ventilation duration in an effort to reduce the incidence of BPD and improve patients’ prognoses.

## Author contributions

Shi X and Li Y were the patient's physician. Shi X and Li Y reviewed the literature and contributed to manuscript drafting; Shi X reviewed the literature and contributed to manuscript drafting; Hua Y conceptualized and designed the study, coordinated and supervised data collection, and critically reviewed the manuscript for important intellectual content. Shi X, Li Y and Hua Y were responsible for the revision of the manuscript for important intellectual content; all authors issued final approval for the version to be submitted.

**Conceptualization:** Xiaoqing Shi, Yimin Hua, Yifei Li.

**Data curation:** Xiaoqing Shi, Yimin Hua, Yifei Li.

**Formal analysis:** Xiaoqing Shi.

**Funding acquisition:** Yimin Hua, Yifei Li.

**Investigation:** Xiaoqing Shi, Yimin Hua, Yifei Li.

**Methodology:** Xiaoqing Shi, Yimin Hua.

**Project administration:** Yimin Hua.

**Supervision:** Yifei Li.

**Validation:** Xiaoqing Shi, Yimin Hua, Yifei Li.

**Visualization:** Yifei Li.

**Writing – original draft:** Xiaoqing Shi, Yifei Li.

**Writing – review & editing:** Yimin Hua, Yifei Li.
